# 

**Published:** 1888-08-11

**Authors:** 


### THE HOSPITAL

A Weekly Institutional Journal of
Science, Medicine, Nursing, and Philanthropy.

### CONTENTS.

**Not Charity, Nurses, but Self-Help** . . . 301

**Aids to Memory** . . . . . . . . 302

**A Defence of the Old Hospital System.** By Sir SYDNEY WATERLOW, Bart., Treasurer of St. Bartholomew's Hospital . 303

**The Science and Art of Housekeeping** . . . 304

**The Doctor's Pulpit.**—"In the Midst of Life" . . 305

**Army Recollections.**—Maiming by Firearms, etc. . . 306

**The Odium Medicum and Homœopathy in America** 306

**In and Out among the Hospitals.**—A Splendid Special Hospital . . . . . . . . 307

**Notes and News** . . . . . . . . 308

**Everybody's Page** . . . . . . . 310

**Annotations.**—Pity the Poor Practitioner.—Child-Cheats.—The Insurance of Children . . . . . . 311

**Physiology and its Uses.**—The Bones of the Hand . . 312

**An Ishmaelite.** By LESLIE KEITH, Author of "The Chilcotes," "East and West," etc. . . . . . . 313

**American Jottings.**—**Questions and Answers** . . 314

**Malingering.**—By W. CURRAN, A.M.D. . . . . 315

**The Student's Column** . . . . . . 316

**Amusements with Profit for all Ages.**—For Men and Women.—Children's Corner.—Puzzles, etc. . . . 316

**EXTRA SUPPLEMENT.—THE NURSING MIRROR.**

En Passant.—French Phrases.—Plain Speaking.—The Sanitary Commission.—The Hamilton Association.—A Small-pox Experience.—Volunteer Nurses . . . . . lxxiii
Original Articles.—A Nurse's Progress.—From Application to Certification.—Paper I. Application . . . lxxiv
British Nurses' Association . . . . . . lxxv
Everybody's Opinion.—District Nursing.—Old Nurses and New.—Seaside Convalescent Homes—Free Training in Midwifery lxxv
Honours and Presentations.—Appointments.—"Quite So."—Notes and Queries.—Vacancies . . . . . . lxxvi

